# A Physics-Informed Hybrid Neural Network for High-Precision Temperature Prediction in Semiconductor Process Equipment

**DOI:** 10.3390/mi17030287

**Published:** 2026-02-25

**Authors:** Jiefeng Peng, Liang Hu, Rui Su, Yingnan Shen, Jing Wang, Xin Fu, Xiaodong Ruan

**Affiliations:** 1 The State Key Laboratory of Fluid Power and Mechatronic Systems, Zhejiang University, Hangzhou 310027, China; 2 Engineering Research Center of DLIS, Ministry of Education, Hangzhou 310058, China

**Keywords:** semiconductor process equipment, precision temperature control, physics-informed machine learning, disturbance prediction

## Abstract

High-precision thermal regulation in semiconductor process equipment is critical for product quality, yet it is challenged by actuator transport delays, limited actuator bandwidth due to hardware dynamics, and broadband inlet disturbances in temperature-controlled process fluids. This paper presents a systematic solution integrating architecture optimization with a physics-informed hybrid prediction model to enable effective feedforward compensation. Frequency-domain analysis justifies placing the temperature fluctuation attenuator (TFA) upstream of the heater to filter mid-to-high-frequency disturbances without compromising feedback stability. To address actuation delays, a Physics-CNN-LSTM predictor is developed using a residual learning strategy. This framework employs a mechanism model for baseline estimation and a deep learning network to correct persistent low-frequency residuals caused by unmodeled dynamics. Comparative experiments on industrial data demonstrate that the model achieves a Root Mean Square Error (RMSE) of $3.56\times 10^{-5}$ K under low-to-mid-frequency inlet disturbances, reducing error by approximately 51.8% compared to a standard LSTM. The model also exhibits strong robustness against disturbance frequency shifts ($R^{2}>0.996$ on unseen data). Furthermore, closed-loop simulations confirm that the proposed feedforward compensation enhances temperature stability in high-precision thermal control.

## 1. Introduction

High-precision temperature regulation is a fundamental requirement in advanced semiconductor process equipment, where temperature-controlled fluids (ultra-pure water) directly affect process stability and product quality. Even minor temperature fluctuations can introduce measurable variations in critical process parameters and degrade overall performance [1,2,3,4,5]. In practice, achieving ultra-stable temperature is challenged by large thermal inertia, actuator delays, multiple disturbance sources, and parameter variations [6,7].

The design of a high-precision temperature control unit (TCU) typically involves two coupled aspects: hardware structure and control strategy. On the hardware side, TFA are widely used to low-pass filter inlet temperature disturbances. Lawton et al. [8,9] analyzed packed-bed and direct-contact attenuator structures and derived transfer functions from heat-transfer principles. More recently, cascaded attenuator configurations have been shown to enhance mid-to-high-frequency attenuation [10], and notch-filter-based designs have also been explored to improve suppression in targeted bands [11]. On the control side, conventional PID feedback can effectively suppress low-frequency fluctuations [12], while advanced strategies such as generalized predictive control, cascade control with feedforward/lag compensation, and MPC-based two-degree-of-freedom schemes have been applied to thermal regulation problems [6,13,14]. Feedforward control is particularly attractive for measurable disturbances and has been reported to improve disturbance suppression in industrial thermal processes [15].

Despite the above progress, the interplay between hardware attenuation and control strategies is often treated separately. In practical operation, the temperature control unit inlet is subjected to low-frequency drifts and mid-to-high frequency fluctuations simultaneously, originating from environmental thermal coupling, load changes, and flow-rate fluctuations. Because heater bandwidth is limited, active feedback predominantly determines performance at low frequencies, whereas passive hardware attenuation becomes dominant at higher frequencies. A frequency-domain viewpoint is therefore useful for allocating regulation mechanisms across disturbance bands and for guiding architecture selection.

For measurable inlet disturbances, feedforward control can further improve performance. However, feedforward design depends on an accurate dynamic model and on advance knowledge of the disturbance. This requirement becomes critical when the actuator exhibits dead time, as is typical for electrical heaters. Although time-delay effects can be approximated by modifying time constants in simplified models [16], practical high-precision feedforward compensation still requires reliable prediction of the disturbance ahead of the actuator delay. Data-driven forecasting methods have thus attracted attention due to their flexibility [17]. Classical machine learning approaches, such as support vector machines (SVMs) [18], extreme gradient boosting (XGBoost) [19], and multilayer perceptrons (MLP) [20], are capable of fitting historical temperature data but often exhibit limited ability to capture long-term temporal dependencies. Recurrent models such as LSTM [21] and GRU [22] have been widely adopted for time-series prediction, and hybrid CNN–LSTM architectures have been reported to enhance feature extraction in forecasting tasks [23].

Hybrid modeling that combines physical principles with data-driven learning provides another approach for temperature prediction. Recent advancements have significantly improved the reliability of deep learning for dynamical systems. To address training stability and ensure reliable weight updating in PDE-related problems, Noorizadegan et al. [24] proposed a power-enhanced residual network (PerNet) that effectively mitigates gradient issues and enhances function approximation accuracy. In parallel, the fusion of recurrent architectures with physical constraints has evolved to handle complex dynamics. For instance, Tao et al. [25] developed a hybrid LSTM-PINN method for steady-state electrohydrodynamic flow, successfully merging temporal learning with physical laws. Similarly, dealing with extreme physical disturbances has been addressed by Xing et al. [26], who utilized interval-constrained PINNs to model dynamic gas–liquid interfaces in underwater explosions, demonstrating the efficacy of constrained learning under severe fluctuation conditions. In such frameworks, a physics-based mechanism model is used to generate a baseline estimate, while the data-driven component accounts for the remaining discrepancy caused by unmodeled dynamics and uncertainties. However, most existing studies focus on coarse-grained temperature prediction problems, such as building HVAC systems [27,28], and their applicability to the high-precision prediction required by semiconductor equipment remains insufficiently explored. In high-precision thermal control applications, the existing solutions are mainly based on feedback regulation, such as active disturbance rejection control (ADRC) [29] and model predictive control (MPC). Prediction-based feedforward disturbance suppression that accounts for actuator dead time is less frequently reported.

This work addresses the above issues by focusing on prediction of the heater-inlet temperature disturbance, which directly supports realizable feedforward compensation. The main contributions are summarized as follows:

- Architecture Optimization and Physical Modeling: A frequency-domain analysis of typical temperature-control architectures is conducted to clarify the roles of active feedback regulation and passive hardware attenuation across disturbance frequency bands. The analysis supports placing the TFA upstream of the heater to pre-filter mid-to-high-frequency inlet disturbances without degrading closed-loop stability. For this configuration, the disturbance-rejection mechanism of adding feedforward compensation is analyzed. A mathematical model of the cascaded TFA is then established to provide a baseline prediction, and residual analysis is performed to characterize discrepancies attributed to unmodeled dynamics.
- Physics-CNN-LSTM Prediction: A Physics-CNN-LSTM predictor employing a residual learning strategy is developed to address the specific challenges of thermal control. By embedding the transport dynamics of the TFA, the proposed framework establishes a deterministic physical baseline essential for industrial safety, effectively mitigating the instability risks associated with “black-box” pure data-driven models. The deep learning module complements this by accurately estimating the nonlinear residuals caused by unmodeled environmental thermal coupling and flow fluctuations, thereby dynamically correcting the physical baseline. Quantitative comparisons against pure data-driven benchmarks—including LSTM, CNN-LSTM, and the advanced CNN-LSTM-Attention model—across distinct operating conditions demonstrate the proposed hybrid model’s superiority in both accuracy and generalization capability. Finally, closed-loop Simulink simulations validate the effectiveness of the proposed feedforward compensation, confirming its feasibility and robustness for high-precision temperature control in advanced semiconductor manufacturing equipment.

## 2. System Analysis and Problem Formulation

### 2.1. System Architecture and Control Strategy Analysis

**Figure 1:**
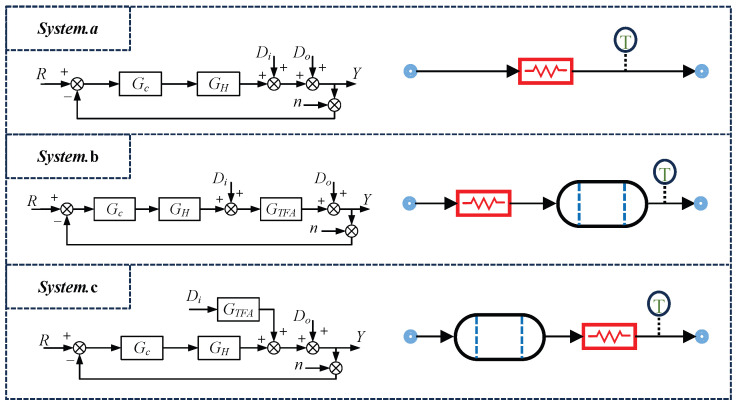
The framework of the control system and the analysis of the control architecture.

To achieve ultra-stable temperature regulation in advanced semiconductor process equipment, the terminal temperature-control unit need to mitigate inlet disturbances over a broad frequency range. As illustrated in Figure 1, three representative functional layouts for the terminal control unit are typically considered: system a, a conventional single-loop feedback control system comprising only an electrical heater; system b, a closed-loop system where a passive TFA is placed downstream of the heater (inside the loop); and system c, an architecture where the TFA is placed upstream of the heater (outside the feedback loop) to act as a pre-filter.

In these configurations, *R*, $D_{i}$, $D_{o}$, *n*, and *Y* denote the reference signal, inlet temperature disturbance, environmental disturbance, measurement noise, and output temperature, respectively. $G_{c}\left(s\right)$, $G_{H}\left(s\right)$, and $G_{TFA}\left(s\right)$ represent the transfer functions of the controller, the heater, and the TFA. Since the inlet disturbance $D_{i}$ is the dominant source of temperature fluctuation, the disturbance rejection capability of each architecture is evaluated using their respective closed-loop disturbance transfer functions from $D_{i}$ to *Y*:

(1) $$S_{a}\left(s\right)=\frac{Y(s)}{D_{i}\left(s\right)}=\frac{1}{1+G_{c}\left(s\right)G_{H}\left(s\right)}$$

(2) $$S_{b}\left(s\right)=\frac{Y(s)}{D_{i}\left(s\right)}=\frac{G_{TFA}\left(s\right)}{1+G_{c}\left(s\right)G_{H}\left(s\right)G_{TFA}\left(s\right)}$$

(3) $$S_{c}\left(s\right)=\frac{Y(s)}{D_{i}\left(s\right)}=\frac{G_{TFA}\left(s\right)}{1+G_{c}\left(s\right)G_{H}\left(s\right)}$$

**Figure 2:**
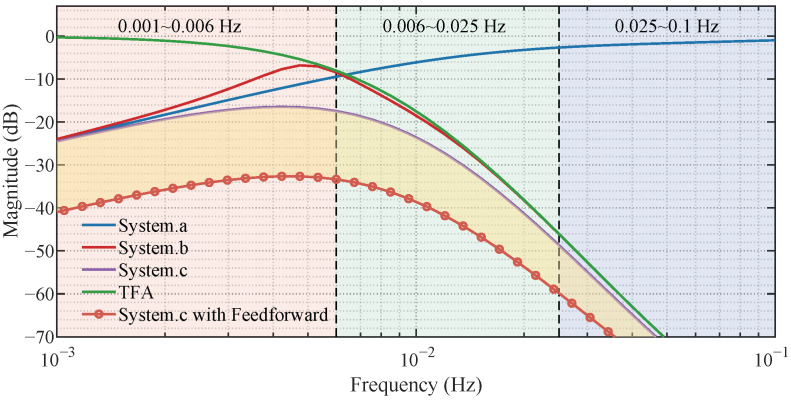
Frequency response comparison of different control architectures.

The frequency responses of these transfer functions are compared in Figure 2 (the magnitude response of the TFA itself is also shown for reference). For clarity, the spectrum is partitioned into three frequency bands using two practical attenuation thresholds of the heater-dominated rejection (system a), i.e., −10 dB and −3 dB. Accordingly, the two boundary frequencies (0.006 Hz and 0.025 Hz) correspond to the points where the rejection magnitude degrades to −10 dB and −3 dB, respectively. In the low-frequency band (0.001–0.006 Hz), feedback regulation via the heater provides effective suppression. As frequency increases, the rejection of system a deteriorates due to the heater bandwidth limitation. Meanwhile, the TFA exhibits a low-pass attenuation behavior over the mid-to-high frequency range; in this work, it is implemented as a cascaded TFA and therefore behaves as a high-order inertial system with transport delay (see Equation (6)).

Although system b attempts to combine active feedback and passive attenuation within the same loop, the large time constants and delay introduced by the TFA significantly reduce the phase margin, which degrades low-frequency suppression. In contrast, system c places the TFA outside the feedback loop, allowing it to pre-filter high-frequency inlet disturbances without introducing additional phase lag into the loop, thereby preserving robust low-frequency rejection.

**Figure 3:**
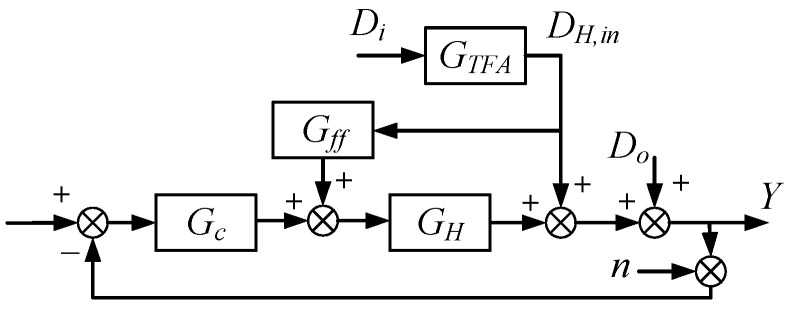
Feedforward control structure.

Nevertheless, feedback control essentially acts as a post-correction mechanism. To further enhance rejection of measurable inlet disturbances, system c is augmented with a feedforward path (Figure 3). A temperature sensor is placed at the heater inlet (i.e., the TFA outlet) to directly measure the attenuated disturbance $D_{H,in}$. A feedforward controller $G_{ff}\left(s\right)$ then generates a compensation signal that is superimposed on the feedback control action, providing pre-compensation before the disturbance propagates to the output. For the feedforward-augmented structure, the closed-loop disturbance transfer can be written as

(4) $$\frac{Y(s)}{D_{i}\left(s\right)}=\frac{\left[1+G_{H}\left(s\right)G_{ff}\left(s\right)\right]G_{TFA}\left(s\right)}{1+G_{c}\left(s\right)G_{H}\left(s\right)}$$

In theory, ideal cancelation of the measurable inlet disturbance can be achieved by choosing $G_{ff}\left(s\right)=-G_{H}^{-1}\left(s\right)$, which makes $1+G_{H}\left(s\right)G_{ff}\left(s\right)=0$. However, the exact inverse requires a time-advance term and a differentiating action, rendering it non-causal and highly sensitive to noise and model uncertainties. Therefore, in practice, $G_{ff}\left(s\right)$ is implemented as a realizable filtered inverse and combined with a 5 s-ahead estimate of $D_{H,in}$, leading to finite (yet significantly improved) attenuation, as shown in Figure 2.

### 2.2. Physical Modeling of Key Components

The efficacy of the aforementioned feedforward control relies heavily on accurate modeling of the system dynamics. The electrical heater, serving as the primary actuator, is identified as a First-Order Plus Dead Time (FOPDT) model using pseudo-random binary sequence (PRBS) excitation. The transfer function is

(5) $$G_{H}\left(s\right)=\frac{K}{T_{H}s+1}\;e^{-\tau_{H}s}=\frac{0.017}{1.6s+1}\;e^{-5s},$$

where *K* is the process gain, $T_{H}$ the time constant, and $\tau_{H}$ the transport delay. Crucially, the heater introduces a pure time delay of $\tau_{H}=5$ s. To achieve perfect feedforward cancelation, the inlet disturbance at the heater must be known at least 5 s in advance. However, this requirement renders direct model-inversion feedforward non-causal in practice (since it would require a time-advance term); therefore, we formulate a 5 s-ahead disturbance prediction problem for $D_{H,in}$ to enable realizable feedforward compensation.

In the proposed architecture, the upstream TFA provides both passive attenuation and a physics-based baseline for predicting the incoming disturbance at the heater inlet. Different from conventional TFA, the TFA considered here adopts a cascaded structure. Building upon the established model of cascaded attenuators [10], the TFA is modeled as

(6) $$\begin{array}{cc} G_{TFA}\left(s\right) & ={\left(\frac{\alpha }{T_{c}s+1}+\frac{\left(1-\alpha \right)\left(1-\beta \right)\frac{1}{T_{m}s+1}}{1-\frac{\beta }{T_{m}s+1}}\right)}^{N+1}e^{-\tau_{TFA}s}\\ \; & ={\left(\frac{1.38s+1}{1.98s^{2}+15.31s+1}\right)}^{6}e^{-6s} \end{array}$$

where α, β, $T_{c}$, and $T_{m}$ characterize the thermal mixing and heat transfer properties. Specifically, α represents the fraction of flow taking the channeling path, β the fraction of the mixture that is recirculated, $T_{c}$ is the bypass time constant, and $T_{m}$ is the mixing time constant. This physical model enables the calculation of a baseline prediction of the TFA outlet temperature (i.e., the heater inlet disturbance $D_{H,in}$) from the measured TFA inlet disturbance $D_{i}$.

### 2.3. Problem Formulation and Residual Analysis

**Figure 4:**
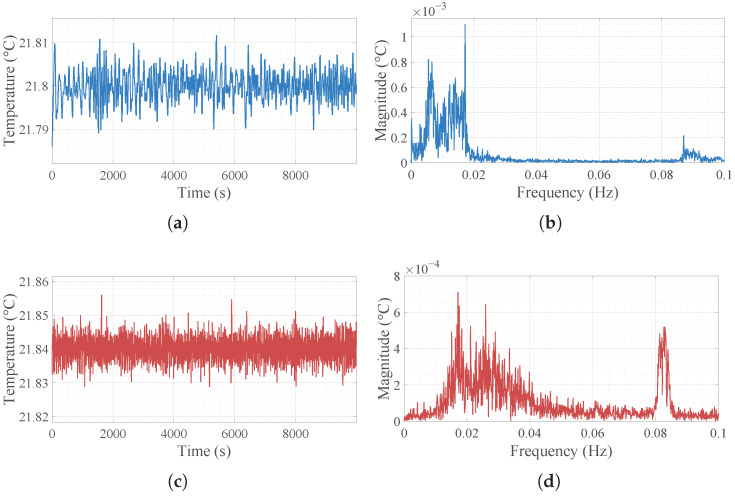
Time-domain and frequency-domain characteristics of the inlet temperature disturbances ($D_{i}$): (**a**) time series under Case 1 (low-to-mid-frequency dominance). (**b**) Frequency spectrum of Case 1. (**c**) Time series under Case 2 (mid-to-high-frequency dominance). (**d**) Frequency spectrum of Case 2.

**Figure 5:**
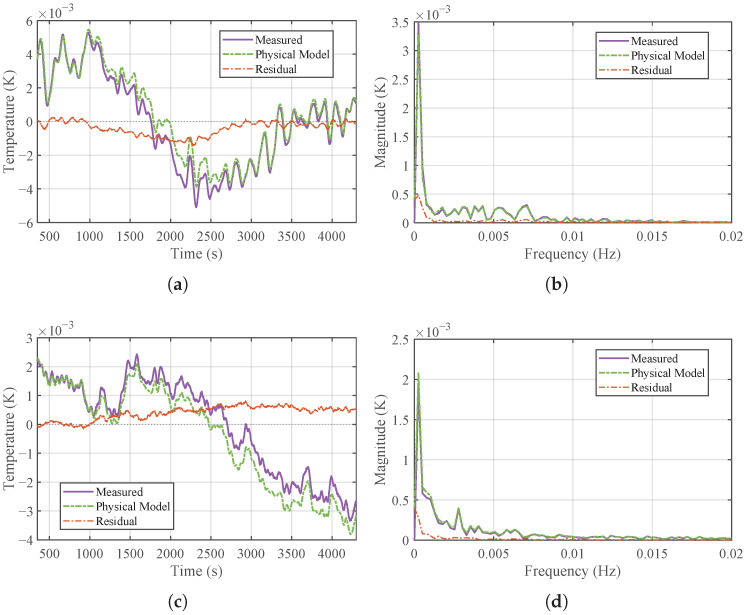
Comparison between physically calculated output and actual measured temperature at the TFA outlet: (**a**) time-domain comparison for Case 1. (**b**) Frequency spectrum of residuals for Case 1. (**c**) Time-domain comparison for Case 2. (**d**) Frequency spectrum of residuals for Case 2.

Although the physical model (Equation (6)) theoretically characterizes the transmission of temperature fluctuations, its real-world performance deviates due to unmodeled dynamics. The time- and frequency-domain characteristics of the inlet disturbances $D_{i}$ under different operating conditions are presented in Figure 4, where two distinct disturbance patterns are observed: one dominated by low-to-mid frequencies and the other by mid-to-high frequencies. Subsequently, a comparison is made between the physically calculated output and the actual measured temperature at the TFA outlet under these categorized disturbance bands, as shown in Figure 5.

The results indicate that while the physical model accurately captures the phase and general trend of the temperature fluctuations (demonstrating high correlation), there exists a persistent prediction error, or residual. As shown in the error curves of Figure 5a,c and further confirmed by its frequency spectrum in Figure 5b,d, this residual is not random white noise but exhibits distinct low-frequency characteristics. This error originates predominantly from unmodeled environmental thermal disturbances, such as ambient heat exchange, and flow rate fluctuations. Consistent with the requirements of high-precision temperature control in semiconductor equipment, a metrology-grade data acquisition system was employed to capture these minute variations. The experimental setup utilized a FLUKE 1594A Super-Thermometer coupled with a FLUKE 5611T precision thermistor sensor. The FLUKE 1594A offers an exceptional instrument accuracy of ±0.0002 °C. Furthermore, to minimize measurement uncertainty, the 5611T thermistor was re-calibrated specifically within a narrow 0.1  °C range relative to the target setpoint, achieving a verified accuracy of ±0.001 °C [30,31].

Consequently, relying solely on the physical model to predict the TFA outlet temperature $D_{H,in}$ (which serves as the heater inlet) based on measured inlet data $D_{i}$ is insufficient for achieving high-precision. The core problem addressed in this study is formulated as follows: given the historical sequence of inlet disturbances and the baseline prediction provided by the physical model, the objective is to accurately predict the residual error, e(t), induced by environmental coupling, in order to reconstruct the precise future temperature at the TFA outlet. An accurate prediction of this future disturbance provides the advance information required by the heater’s feedforward controller to compensate for its inherent actuation delay, which is essential for enabling high-precision temperature control.

## 3. Physics-Informed Hybrid Framework

### 3.1. Hybrid Prediction Architecture

To overcome the limitations of purely analytical models, a hybrid prediction framework is developed by integrating the physical mechanism of the TFA with a data-driven deep learning architecture. This model is conceptualized as a nonlinear mapping function that takes observed sequence values over a period as input to predict the future disturbances at the heater inlet.

**Figure 6:**
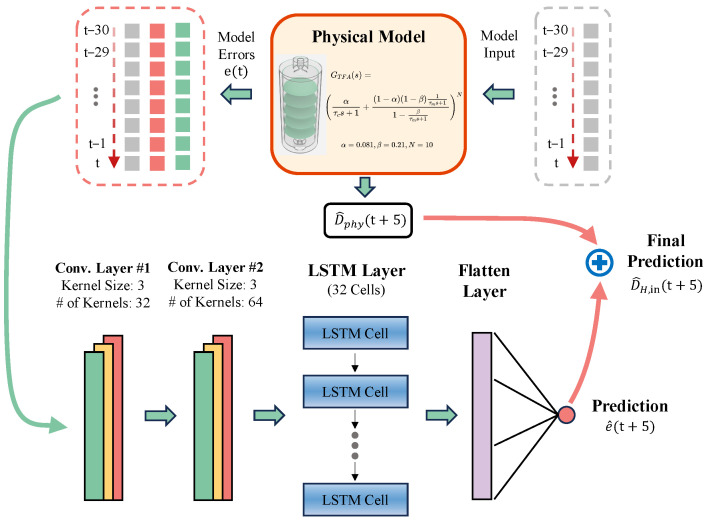
Architecture of the Physics-CNN-LSTM hybrid model.

The overall architecture, illustrated in Figure 6, adopts a residual learning strategy. Instead of predicting the absolute temperature disturbance directly, the neural network is designed to estimate the discrepancy, or residual, between the physical model’s output and the actual measured values. $D_{i}\left(t\right)$ denotes the inlet temperature disturbance of the TFA and $D_{H,in}\left(t\right)$ denote the actual disturbance at the TFA outlet (which corresponds to the heater inlet). The physical model, driven by $D_{i}\left(t\right)$, provides an estimated baseline ${\hat{D}}_{phy}\left(t\right)$. The residual is defined as

(7) $$e\left(t\right)=D_{H,in}\left(t\right)-{\hat{D}}_{phy}\left(t\right)$$

The hybrid predictor utilizes historical observations to estimate the future residual $\hat{e}\left(t+5\right)$. The final 5 s ahead prediction is obtained by combining the physical baseline and the predicted residual:

(8) $${\hat{D}}_{H,in}\left(t+5\right)={\hat{D}}_{phy}\left(t+5\right)+\hat{e}\left(t+5\right)$$

This approach ensures that the model remains grounded in physical conservation laws while leveraging the ability of neural networks to capture unmodeled nonlinearities, such as ambient heat exchange and flow fluctuations.

### 3.2. Network Design and Input Representation

To enhance sensitivity to the slowly varying thermal drift induced by ambient coupling and flow fluctuations, the model input is constructed as a multi-dimensional feature sequence that includes both raw measurements and physics-derived signals.

At each time instant *t*, the feature vector is defined as

(9) $$x\left(t\right)={\left[\;D_{i}\left(t\right),\;{\hat{D}}_{phy}\left(t\right),\;e\left(t\right),\;\Delta e\left(t\right)\;\right]}^{⊤}$$

where Δe(t)=e(t)−e(t−1) is the first-order difference of the residual, characterizing the instantaneous change rate of the unmodeled thermal gradient.

Using a sliding window of length *L*, the network input at time *t* is

(10) $$X_{t}=\left[\;x(t-L+1),\;x(t-L+2),\;\ldots ,\;x(t)\;\right]$$

and the learning target is the future residual e(t+5). Consequently, the neural network implements the nonlinear mapping

(11) $$\hat{e}\left(t+5\right)=N_{\theta }\left(X_{t}\right)$$

where $N_{\theta }\left(\cdot \right)$ denotes the CNN–LSTM predictor parameterized by θ.

The detailed architecture configuration of the CNN-LSTM predictor $N_{\theta }$ is designed to process the input matrix $X_{t}$, where the window length *L* is set to 30. Two convolutional layers are stacked to extract local nonlinear patterns, with the number of kernels set to 32 and 64, respectively. These hyperparameters were determined through empirical experiments to balance feature extraction performance with computational efficiency. A uniform kernel size of 3 is applied in both layers using ReLU activation functions. All convolutional operations are implemented with a TimeDistributed layer to preserve temporal dimensions [32,33]. After utilizing the local feature extraction capability of the convolutional layers, the resulting feature maps are flattened and introduced to the LSTM component to process the temporal progression of the data. An LSTM layer comprising 32 hidden units is employed to interpret the long-term dependencies within the sequential data. Finally, the output is passed through a fully connected layer to generate the residual prediction.

## 4. Experimental Results and Discussion

### 4.1. Experimental Setup, Data Preprocessing, and Metrics

To validate the effectiveness of the proposed Physical-CNN-LSTM framework, experiments were conducted using high-precision temperature data sampled from the actual TFA outlet data. The dataset includes two distinct operating conditions with varying disturbance characteristics, allowing us to assess both prediction accuracy and model robustness. The proposed hybrid model is compared against three data-driven benchmarks: a standard LSTM network, a CNN-LSTM network, and a CNN-LSTM-Attention network [34]. The latter integrates an attention mechanism following the LSTM layer to highlight critical temporal features. For a fair comparison with purely data-driven predictors, all three baselines use only historical measurements of the heater-inlet disturbance and its first-order difference. The baseline feature vector is defined as

(12) $$x_{b}\left(t\right)={\left[D_{H,in}\left(t\right),\;\Delta D_{H,in}\left(t\right)\right]}^{⊤},\;\Delta D_{H,in}\left(t\right)=D_{H,in}\left(t\right)-D_{H,in}\left(t-1\right).$$

Using the same sliding window length L=30, the baseline input matrix at time *t* is

(13) $$X_{t}^{\left(b\right)}=\left[x_{b}\left(t-L+1\right),\ldots ,x_{b}\left(t\right)\right],$$

and the prediction target is $D_{H,in}\left(t+5\right)$ (equivalently, the 5s ahead disturbance).

Data Preprocessing: To ensure numerical stability during training and mitigate issues such as gradient explosion or vanishing, data normalization is essential. Z-score standardization is applied to all input sequences. Each feature *x* is normalized using the following formula:

(14) $$x_{\mathrm{norm}}=\frac{x-\mu }{\sigma }$$

where μ and σ denote the mean and standard deviation.

Performance Metrics: The predictive performance is quantitatively evaluated using three standard metrics: Mean Absolute Error (MAE), Root Mean Square Error (RMSE), and the Coefficient of Determination ($R^{2}$). The mathematical expressions for these metrics are as follows:

(15) $$\mathrm{MAE}=\frac{1}{N}\sum_{i=1}^{N}\left|D_{H,in}\left(t\right)-{\hat{D}}_{H,in}\left(t\right)\right|$$

(16) $$\mathrm{RMSE}=\sqrt{\frac{1}{N}\sum_{i=1}^{N}{\left(D_{H,in}\left(t\right)-{\hat{D}}_{H,in}\left(t\right)\right)}^{2}}$$

(17) $$R^{2}=1-\frac{\sum_{i=1}^{N}{\left(D_{H,in}\left(t\right)-{\hat{D}}_{H,in}\left(t\right)\right)}^{2}}{\sum_{i=1}^{N}{\left(D_{H,in}\left(t\right)-{\bar{D}}_{H,in}\right)}^{2}}$$

where ${\bar{D}}_{H,in}$ represents the mean of the observed values.

To ensure the reliability of the experimental results and mitigate the impact of randomness caused by weight initialization, all models (including the proposed method and baselines) are trained and evaluated independently 10 times using different random seeds. The final performance metrics are reported as the mean ± standard Ddeviation of these 10 runs.

### 4.2. Analysis of Results

**Figure 7:**
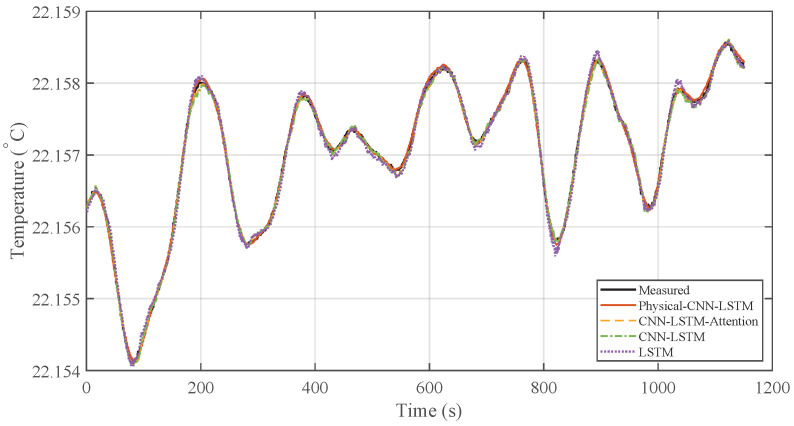
Prediction results comparison under Case 1.

**Figure 8:**
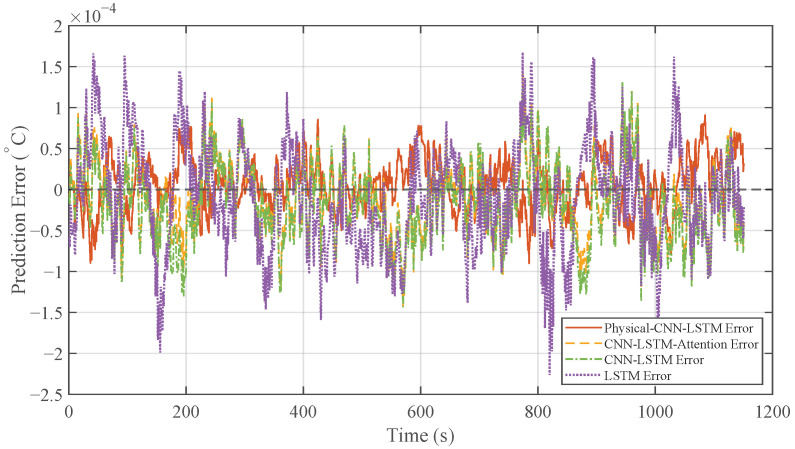
Comparison of prediction errors under Case 1.

To evaluate the performance of the proposed Physical-CNN-LSTM model in predicting the heater inlet temperature ($D_{H,in}$), the LSTM, CNN-LSTM, and CNN-LSTM-Attention models were selected as benchmarks. The prediction results under the low-to-mid-frequency disturbance condition (Case 1) are illustrated in Figure 7, with the corresponding prediction errors presented in Figure 8.

As observed from the time-domain comparison in Figure 7, the prediction curve of the Physical-CNN-LSTM model aligns most closely with the actual measured data, while all four models follow the general trend, the LSTM, CNN-LSTM, and CNN-LSTM-Attention models exhibit larger deviations, particularly at local peaks and valleys where the disturbance changes rapidly. In contrast, the Physical-CNN-LSTM model demonstrates improved tracking accuracy. This is further reflected in the error curves in Figure 8, where the proposed model exhibits a smaller fluctuation amplitude, oscillating near the zero-error line, whereas the single LSTM model displays the largest error magnitude.

**Figure 9:**
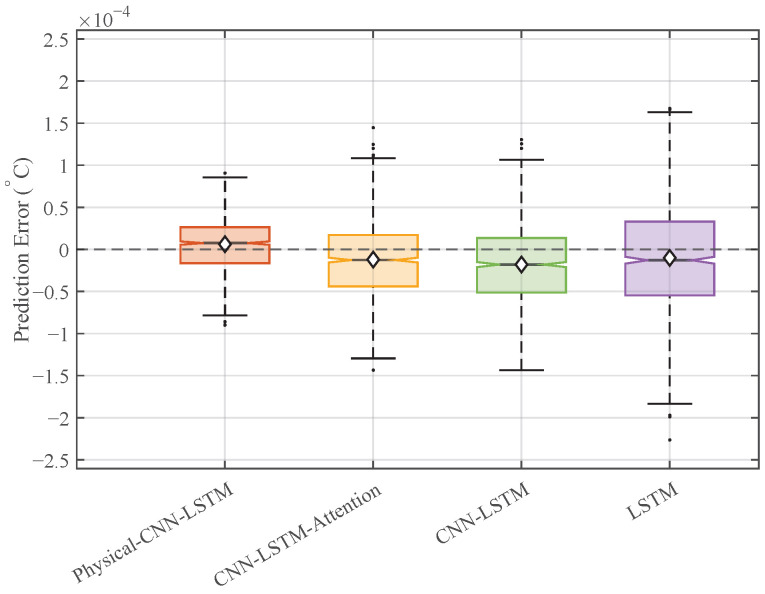
Box plot distribution of prediction errors under Case 1.

Figure 9 presents the distribution of prediction errors. The Physical-CNN-LSTM model demonstrates a narrower error range compared to the benchmarks, with the data points concentrated near the zero line. The median alignment with zero suggests a low level of systematic bias. In contrast, the box plots for the LSTM, CNN-LSTM, and CNN-LSTM-Attention models exhibit a wider spread and a greater number of outliers, indicating higher variability when handling dynamic thermal fluctuations.

To quantify the prediction performance, the values of the evaluation metrics (MAE, RMSE, $R^{2}$) for the four models are listed in Table 1. It is evident that the proposed Physical-CNN-LSTM framework achieves superior prediction accuracy, significantly outperforming all three data-driven baselines. Specifically, the Physical-CNN-LSTM model yields an RMSE of $3.56\pm 0.24\times 10^{-5}$ K. This represents a substantial error reduction of approximately 31.8% compared to the CNN-LSTM-Attention model ($5.22\pm 0.42\times 10^{-5}$ K), 36.9% compared to the CNN-LSTM ($5.64\pm 0.46\times 10^{-5}$ K), and 51.8% relative to the LSTM model ($7.39\pm 1.04\times 10^{-5}$ K). Regarding the MAE, the proposed method achieves a reduction of 51.0% compared to the LSTM model. Furthermore, the high coefficient of determination ($R^{2}=0.99876\pm 0.0002$) confirms the model’s exceptional precision and stability.

### 4.3. Generalization and Robustness Analysis

To assess the generalization capability and robustness of the proposed framework, the model trained on Case 1 was directly applied to predict the temperature in two distinct unseen scenarios (Case 2 and Case 3) without any retraining. Both Case 2 and Case 3 represent scenarios dominated by mid-to-high-frequency inlet disturbances ($D_{i}$).

**Figure 10:**
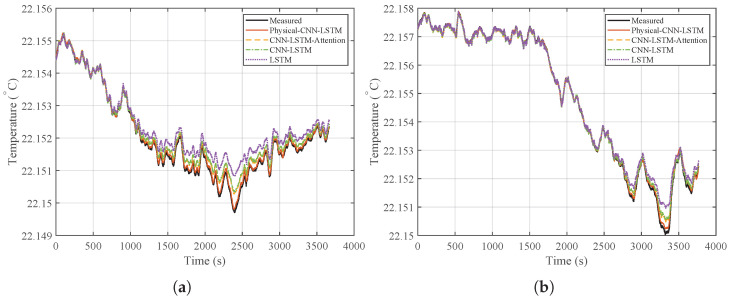
Generalization performance comparison: (**a**) prediction results under Case 2 (mid-to-high frequency disturbances). (**b**) Prediction results under Case 3 (additional mid-to-high frequency disturbances).

**Figure 11:**
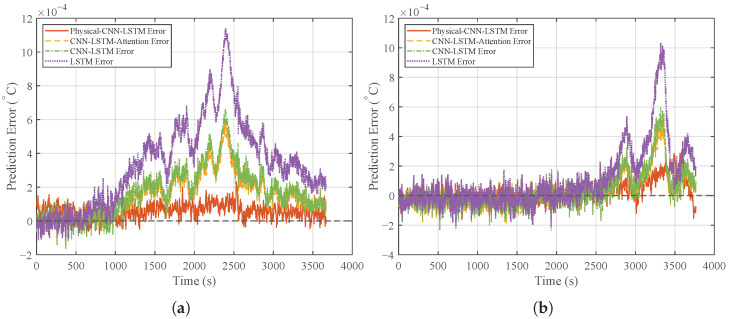
Generalization performance comparison: (**a**) prediction errors under Case 2. (**b**) Prediction errors under Case 3.

The prediction results and the corresponding error curves for this generalization test are shown in Figure 10 and Figure 11, respectively. Figure 10 shows that the Physical-CNN-LSTM model maintains the closest tracking performance, followed by CNN-LSTM-Attention, while the LSTM model exhibits the largest deviation. Even under shifts in disturbance frequency (Case 2 and Case 3), the proposed model continues to track the true values. In contrast, the LSTM model shows noticeable amplitude distortion, leading to increased residual errors as shown in Figure 11.

**Figure 12:**
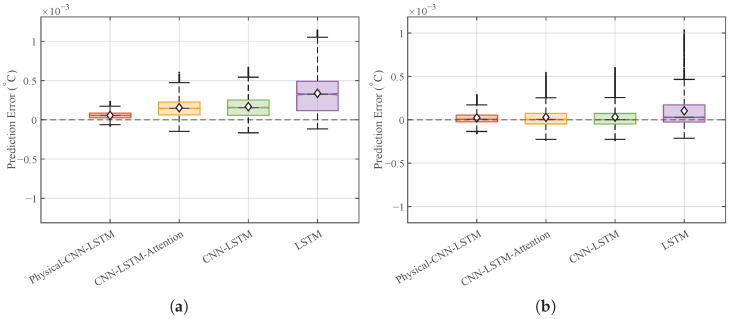
Generalization performance comparison: (**a**) box plot distribution of prediction errors under Case 2. (**b**) Box plot distribution of prediction errors under Case 3.

Figure 12 reveals a clear difference in stability. The Physical-CNN-LSTM model preserves a narrow and concentrated error distribution, similar to that in Case 1. Conversely, the benchmark models exhibit a much wider spread, reflecting increased variability. This indicates that while pure data-driven models struggle to generalize to mid-to-high-frequency inlet disturbances in the TFA, the hybrid model remains robust due to the constraints provided by its physical component.

Table 2 summarizes the quantitative performance on the unseen datasets (Case 2 and Case 3). In Case 2, dominated by mid-to-high-frequency disturbances, the LSTM model shows a marked performance decline, with its $R^{2}$ dropping to 0.80254±0.0572. In contrast, the Physical-CNN-LSTM maintains a high $R^{2}$ of 0.99535±0.0014 and achieves an RMSE of $8.92\pm 1.36\times 10^{-5}$ K, reducing the error by 84.6% compared to the LSTM baseline. This robustness is further confirmed in Case 3, where the proposed model again records the lowest RMSE ($8.33\pm 0.85\times 10^{-5}$ K) and the highest $R^{2}$ (0.9988±0.0002). These results indicate that incorporating physical constraints helps maintain prediction accuracy across different operating frequencies and conditions.

Impact of Prediction Horizon: Beyond the disturbance frequency, the model’s performance is intrinsically correlated with the actuator delay length (prediction horizon). In scenarios with a shorter delay (e.g., <2 s), the stronger temporal correlation between historical inputs and future states would theoretically result in higher prediction accuracy. Conversely, a significantly longer delay (e.g., >10 s) would introduce greater uncertainty. However, the proposed hybrid architecture utilizes the deterministic physical baseline as a constraint, thereby maintaining greater robustness.

### 4.4. Ablation Study

To quantify the contributions of the physical baseline and the specific network components, an ablation study was conducted across three distinct cases. Four model configurations were evaluated, Pure Physical Model, Pure CNN-LSTM, Physical-LSTM (w/o CNN), and the proposed method, with all performance results summarized in Table 3.

As observed in Table 3, the Pure Physical Model exhibits the highest RMSE across all cases, indicating that the theoretical model alone cannot capture the complex unmodeled dynamics and environmental disturbances. However, its role becomes important when comparing the Pure CNN-LSTM with the Physical-LSTM. In Case 2, the Pure CNN-LSTM suffers a performance degradation with an RMSE of 24.2 ± 2.08. In contrast, by simply incorporating the physical baseline, the Physical-LSTM (w/o CNN) reduces the RMSE to 10.3 ± 0.89, a reduction of approximately 57.4%. This result confirms that the physical baseline provides a stabilizing constraint, preventing the neural network from overfitting to specific frequency patterns and ensuring robustness in unseen scenarios. The comparison between Physical-LSTM and the proposed Physical-CNN-LSTM highlights the specific contribution of the convolutional layers. Across all three cases, the inclusion of the CNN module consistently lowers the prediction error.

### 4.5. Closed-Loop Control Simulation Validation

To validate the practical effectiveness of the proposed prediction model in a real-world control scenario, a closed-loop simulation was conducted. The objective is to demonstrate that the high-precision prediction of heater-inlet disturbances ($D_{H,in}$) translates into temperature stability compared to conventional feedback control.

A simulation environment was constructed in Simulink/MATLAB R2024b to replicate the dynamics of the experimental heating system. The disturbance signals from Cases 1–3 described previously were utilized as the inlet disturbance inputs ($D_{H,in}$) for the simulation. The control loop incorporates the heater plant model $G_{H}\left(s\right)$ identified in Equation (5), characterized by a critical 5-second transport delay, alongside a standard PID controller tuned for baseline stability following standard tuning guidelines [35,36] (with parameters set to $K_{p}=20$ and $K_{i}=6$). Crucially, the predicted future disturbance ${\hat{D}}_{H,in}\left(t+5\right)$, generated by the proposed Physical-CNN-LSTM model, is injected into the control loop as a feedforward compensation signal. To ensure physical realizability, the feedforward controller is implemented as the inverse of the plant model cascaded with a first-order low-pass filter, formulated as

(18) $$G_{ff}\left(s\right)=G_{H}^{-1}\left(s\right)\cdot \frac{1}{0.1s+1}$$

**Figure 13:**
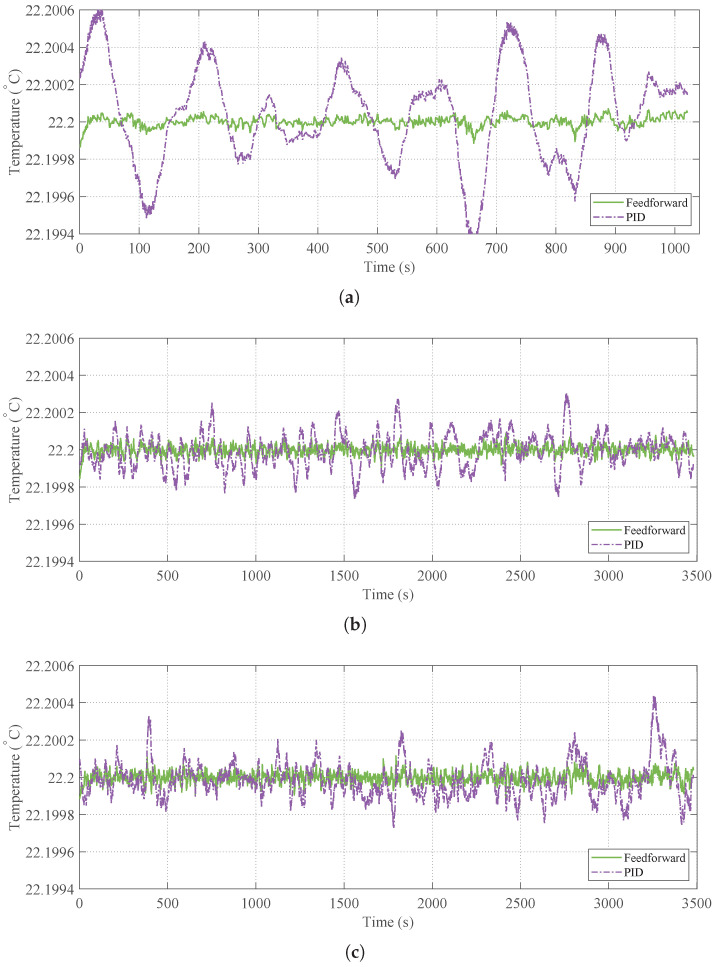
Closed-loop temperature regulation performance comparing conventional PID feedback with the proposed Feedforward–PID strategy: (**a**) control response under Case 1. (**b**) Control response under Case 2. (**c**) Control response under Case 3.

The temperature regulation performance under the three distinct disturbance cases is compared in Figure 13. The conventional PID controller and the proposed feedforward–PID strategy are evaluated against the target setpoint (22.20 °C).

As illustrated in Figure 13a (Case 1), the inlet disturbance frequencies are concentrated in the 0.006–0.025 Hz band, approaching the physical bandwidth limit of the heater where actuation performance degrades with increasing frequency. The conventional PID controller, constrained by its inherent feedback nature and the transport delay, fails to effectively suppress these disturbances. In contrast, the proposed feedforward strategy utilizes the 5 s-ahead prediction to preemptively cancel the disturbance impact, resulting in a significantly smoother temperature profile. Regarding Case 2 and Case 3 (Figure 13b,c), although the inlet disturbances exhibit higher-frequency components, their amplitudes are relatively small, and the spectrum remains dominated by low-frequency components. Consequently, while the PID controller can partially suppress the dominant low-frequency disturbances, it struggles to mitigate high-frequency sudden fluctuations. However, the Physical-CNN-LSTM model accurately captures both the global trends and detailed fluctuations. By injecting this comprehensive prediction into the feedforward path, the system achieves a near-flat response, further reducing temperature fluctuations compared to the PID baseline.

## 5. Conclusions

This paper addressed the challenge of high-precision temperature control in semiconductor process equipment by conducting a systematic analysis of system architectures and developing a physics-informed hybrid prediction model.

First, a frequency-domain analysis of typical terminal temperature-control architectures was conducted to clarify the complementary roles of active feedback regulation and passive hardware attenuation across different disturbance frequency bands. Based on this analysis, an architecture placing the temperature fluctuation attenuator (TFA) upstream of the heater was identified as a robust solution, because it pre-filters mid-to-high-frequency inlet disturbances ($D_{i}$) without degrading the feedback loop stability. Moreover, realizable feedforward compensation is fundamentally constrained by the heater’s transport delay, which requires reliable advance knowledge of the heater-inlet disturbance ($D_{H,in}$). Residual analysis on experimental data further showed that, although the physical model captures the overall trend and phase of temperature fluctuations, a persistent low-frequency residual remains due to unmodeled environmental heat exchange and flow-rate fluctuations. Therefore, a higher-precision prediction approach is required to provide accurate future disturbance estimates for effective feedforward compensation.

Consequently, a Physics-CNN-LSTM hybrid framework was proposed. This model utilizes the physical mechanism to establish a baseline while employing a deep learning network to accurately compensate for the residual errors. Experimental results based on industrial data lead to the following conclusions:

- Prediction Accuracy (Case 1): Under low-to-mid-frequency disturbance conditions, the Physical-CNN-LSTM model achieved an RMSE of $3.56\times 10^{-5}$ K and an $R^{2}$ of 0.99876. Compared to the standard LSTM model, the prediction error was reduced by approximately 51.8%.
- Generalization Performance (Case 2, 3): When tested against unseen mid-to-high-frequency disturbances without retraining, the hybrid model maintained an $R^{2}>0.996$. In contrast, pure data-driven models showed performance degradation. These findings indicate that embedding physical constraints effectively prevents overfitting and improves robustness.

Simulation results validated the practical utility of the proposed method. Integrating the high-precision predictor into a feedforward–PID control loop successfully suppressed temperature fluctuation peaks caused by inlet disturbances, achieving a more stable temperature profile compared to conventional feedback control.

These findings indicate that embedding physical constraints improves prediction stability across disturbance-spectrum shifts and reduces overfitting to specific training patterns, which is critical for supporting high-precision feedforward temperature control.

Future work will focus on closed-loop validation by integrating the proposed predictor into a practical feedforward controller and quantifying the resulting improvement in temperature stability. Additionally, considering the recent advancements in neural network architectures, we plan to explore Kolmogorov–Arnold Networks (KANs) as a potential alternative to the CNN-LSTM residual block. KANs have shown promise in physics-informed modeling and may offer further improvements in parameter efficiency and interpretability for dynamical system approximation.

## Figures and Tables

**Table 1:** Quantitative comparison of prediction performance under Case 1.

Method	MAE ($10^{-5}$)	RMSE ($10^{-5}$)	$R^{2}$
LSTM	5.86 ± 0.74	7.39 ± 1.04	0.99455 ± 0.0016
CNN-LSTM	4.54 ± 0.36	5.64 ± 0.46	0.99686 ± 0.0005
CNN-LSTM-Attention	4.24 ± 0.37	5.22 ± 0.42	0.99731 ± 0.0004
Physical-CNN-LSTM	2.87±0.19	3.56±0.24	0.99876±0.0002

**Table 2:** Quantitative generalization performance on unseen datasets (Case 2 and Case 3).

Method	MAE ($10^{-5}$)	RMSE ($10^{-5}$)	$R^{2}$
***Case 2***			
LSTM	47.8 ± 7.75	58.1 ± 8.72	0.80254 ± 0.0572
CNN-LSTM	19.3 ± 1.70	24.2 ± 2.08	0.96631 ± 0.0061
CNN-LSTM-Attention	18.7 ± 1.36	23.2 ± 1.64	0.96902 ± 0.0043
Physical-CNN-LSTM	7.69±1.26	** 8.92±1.36 **	0.99535±0.0014
***Case 3***			
LSTM	19.0 ± 2.95	32.1 ± 4.79	0.98190 ± 0.0005
CNN-LSTM	9.12 ± 0.56	13.9 ± 1.01	0.99662 ± 0.0005
CNN-LSTM-Attention	8.79 ± 0.41	13.4 ± 0.77	0.99691 ± 0.0003
Physical-CNN-LSTM	5.78±0.49	8.33±0.85	0.99879±0.0002

**Table 3:** Ablation study of the proposed hybrid framework under varying operating conditions.

Method	MAE ($10^{-5}$)	RMSE ($10^{-5}$)	$R^{2}$
***Case 1***			
Pure Physical Model	41.72	55.43	0.95729
Pure CNN-LSTM	4.54 ± 0.36	5.64 ± 0.46	0.99686 ± 0.0005
Physical-LSTM (w/o CNN)	3.09 ± 0.24	3.83 ± 0.29	0.99855 ± 0.0002
Physical-CNN-LSTM	2.87±0.19	3.56±0.24	0.99876±0.0002
***Case 2***			
Pure Physical Model	12.29	14.31	0.98814
Pure CNN-LSTM	19.3 ± 1.70	24.2 ± 2.08	0.96631 ± 0.0061
Physical-LSTM (w/o CNN)	8.72 ± 0.87	10.3 ± 0.89	0.99383 ± 0.0011
Physical-CNN-LSTM	7.69±1.26	8.92±1.36	0.99535±0.0014
***Case 3***			
Pure Physical Model	33.14	46.49	0.96284
Pure CNN-LSTM	9.12 ± 0.56	13.9 ± 1.01	0.99662 ± 0.0005
Physical-LSTM (w/o CNN)	7.79 ± 0.23	12.9 ± 0.49	0.99709 ± 0.0002
Physical-CNN-LSTM	5.78±0.49	8.33±0.85	0.99879±0.0002

## Data Availability

The data presented in this paper are available on request from the corresponding author. The data are not publicly available due to privacy restrictions.
